# Expérience oto-rhino-laryngologique de l'hôpital marocain de campagne en Guinée Conakry

**DOI:** 10.11604/pamj.2014.19.40.4908

**Published:** 2014-09-18

**Authors:** Hicham Attifi, Mounir Hmidi, Ali Boukhari, Nabil Touihem, Mounir Kettani, Mohammed Zalagh, Abdelmjid Messary

**Affiliations:** 1 Service d'Oto-Rhino-Laryngologie, Hôpital Militaire Moulay Ismail, Meknès, Maroc

**Keywords:** Affections ORL, regroupement diagnostique, regroupement topographique, ENT disorders, diagnostic grouping, topographic clustering

## Abstract

Il s'agit d'une étude prospective et descriptive portant sur les patients admis en consultation d'oto-rhino-laryngologie, au sein de l'hôpital marocain médico-chirurgical de campagne, déployé en Guinée Conakry, du 24 février au 24 mai 2014. Les critères d'inclusion étaient l’âge, le sexe, le type et la localisation de l'affection. L'objectif de notre étude est d’établir un regroupement diagnostique et topographique des principales affections otorhinolaryngologiques rencontrées en Guinée Conakry. Ont été examinés 1877 patients, soit une incidence de 8,14% si l'on rapporte au nombre total de la consultation pluridisciplinaire. La série comprenait 56,04% de femmes (n =1052) et 43,96% d'hommes (n = 825). L’âge de nos patients variait de 9 mois à 73 ans avec une moyenne de 33 ans, la tranche d’âge la plus touchée était celle de 20 ans à 29 ans et 53,54% des patients avaient un âge compris entre 20 ans et 39 ans. Au plan diagnostique, la pathologie infectieuse était la plus fréquente (54,51%), suivie de la pathologie ototologique non infectieuse et non tumorale entrainant surdité ou acouphènes (18,96%), de la pathologie inflammatoire rhino-sinusienne et pharyngée (18,01%), de la pathologie tumorale (6,34%), des corps étrangers de la sphère ORL (0,69%), des chéloïdes (0,43%) et de la pathologie malformative (0,37%). Au plan topographique, les affections rhino-sinusiennes étaient les plus fréquentes (37,93%), suivies des affections otologiques (33,46%), des affections oro-pharyngées et laryngées (21,20%) et des affections cervicales (6,34%).

## Introduction

Dans le cadre de la coopération militaire Maroco-Guinéenne, le Maroc a déployé en février 2014, un hôpital médico-chirurgical de campagne à Conakry pour une action humanitaire, dont le but était de dispenser des prestations médicales et chirurgicales au profit de la population. Les affections otorhinolaryngologiques en Afrique subsaharienne sont riches et variées. Leur prévention et leur prise en charge sont basés sur l'analyse des données épidémiologiques locales [1]. Peu d’études ont abordé la description des affections otorhinolaryngologiques (ORL) en Guinée Conakry. Ce travail est une étude descriptive et prospective, sous forme d'un bilan de consultation otorhinolaryngologique. Son but est d’établir un regroupent diagnostique et topographique des principales affections ORL chez la population de Guinée Conakry.

## Méthodes

**Figure 1:**
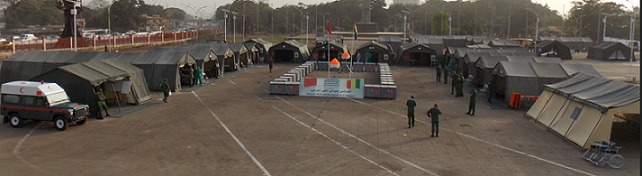
Site d'implantation de l'Hôpital Militaire Médico-chirurgical de compagne en Guinée-Conakry

Il s'agit d'une étude descriptive et prospective, portant sur les patients admis en consultation d'otorhinolaryngologie, au sein de l'hôpital militaire médico-chirurgical de campagne (HMCC) marocain, déployé en Guinée Conakry, du 24 février au 24 mai 2014. La Guinée Conakry est un pays de l'Afrique sub-saharienne, borné par l'océan atlantique. La population est de 10 211437 habitants. Conakry, la capitale, compte environ 2 500000 habitants. L'HMCC est une unité mobile projetable en tout lieu et en toute circonstance, dotée de 11 modules de diagnostic et de prise en charge pour les consultations spécialisées (médecine interne, cardiologie, pneumologie, pédiatrie, ophtalmologie, otorhinolaryngologie, gynécologie, chirurgie viscérale et orthopédique, odontologie et dermatologie ) et de 4 modules constituant le plateau technique avec un laboratoire polyvalent, une unité de radiologie conventionnelle et d’échographie, un bloc opératoire et une pharmacie pour la distribution gratuite des médicaments (Figure 1). Le staff technique était composé de 19 médecins, 2 pharmaciens, 26 infirmiers et 3 secrétaires médicaux. Les actions de L'HMCC étaient coordonnées avec les services médicaux des Forces Armées Guinéennes et le Centre Hospitalier Universitaire (CHU) de Conakry. Les patients reçus en consultation d'otorhinolaryngologie ont bénéficié d'un interrogatoire précisant l’âge, le sexe et les antécédents pathologiques particuliers et d'un examen otorhinolaryngologique complet avec notamment un examen sous miroir de clar de la cavité Buccale et de l'oropharynx, une rhinoscopie antérieure et postérieure, une laryngoscopie indirecte, une otoscopie avec acoumétrie instrumentale et un examen cervical. Les examens para-cliniques sollicités étaient réalisés, soit au niveau de notre structure, soit au niveau du CHU de Conakry.

## Résultats

Durant la période d’étude, 23068 prestations médicales étaient données, 1877 patients étaient admis en consultation ORL, soit une incidence de 8,14 % si l'on rapporte au nombre total de la consultation pluridisciplinaire. La série comprenait 56,04% de femmes (n = 1052) et 43,96% d'hommes (n =825). L’âge de nos patients variait de 9 mois à 73 ans avec une moyenne de 33 ans, la tranche d’âge la plus touchée était celle de 20 ans à 29 ans et 53,54% des cas avaient un âge compris entre 20 ans et 39 ans (Tableau 1). Sur le plan diagnostique, la pathologie infectieuse était la plus fréquente (54,51%), suivie de la pathologie ototologique non infectieuse et non tumorale entrainant surdité ou acouphènes (18,96%), de la pathologie inflammatoire rhino-sinusienne et pharyngée (18,01%), de la pathologie tumorale (6,34%), des corps étrangers de la sphère orl (0,69%), des chéloïdes (0,43 %) et de la pathologie malformative (0,37%) (Tableau 2). Au plan topographique, les affections rhino-sinusiennes étaient les plus fréquentes (37,93%), suivie des affections otologiques (33,46%), des affections oro-pharyngées et laryngées (21,20%) et des affections cervicales (6,34%). A noter que la topographie de 1,07% des affections était non précisée ou concernait plusieurs sites de la sphère ORL (Tableau 3).

**Tableau 1:** Répartition des patients en fonction des tranches d’âge

Tranches d’âge (par an)	Nombre de cas	Pourcentage en (%)
0_9	94	5,01
10_19	190	10,12
20_29	533	28,39
30_39	472	25,15
40_49	350	18,65
50_59	170	9,06
60_69	60	3,20
70_79	8	0,42
Total	1877	100

**Tableau 2:** Regroupement diagnostique des principales affections ORL

Pathologies		Nombre de cas	Fréquence(%)
Pathologies infectieuses :		1023	54,51
	Rhino-sinusienne	377	20,09
	Pharyngo-laryngée	375	19,98
	Otologique	262	13,96
	Ganglionnaire	9	0,48
Pathologies otologiques entrainant Surdité ou acouphènes :		356	18,96
Pathologie inflammatoire rhino-sinusienne et pharyngée		338	18,01
	Pharyngites chroniques	10	0,53
	Rhinites chroniques	281	14,97
	Rhinosinusites chroniques	47	2,51
Pathologie tumorale		119	6,34
	Thyroïdienne	104	5,54
	Pharyngo-laryngée	7	0,37
	Salivaire	6	0, 32
	Massif facial	2	0,11
Corps étrangers de la sphère ORL		13	0,69
Chéloïdes		8	0,43
Pathologie malformative		7	0,37
	Fentes labiales	4	0,21
	Malformations vasculaires	2	0,11
	Agénésie de l'oreille	1	0,05
Autres		13	0,69
	Paralysies faciales	4	0,21
	Traumatismes de la sphère ORL	1	0,05
	Algies divers	6	0,32
	Non précisé	2	0,11
Total		1877	100

**Tableau 3:** Regroupement topographique des principales affections ORL

Topographie	Nombre de cas	Pourcentage
Rhino-sinusienne	712	37 ,93
Otologique	628	33 ,46
Oro-Pharyngo-Laryngée	398	21,20
Cervicale	119	6,34
Multiple ou non précisée	20	1,07
Total	1877	100

## Discussion

### Spécificités épidémiologiques

La consultation otorhinolaryngologique représentait 8,14% de l'ensemble des consultations pluridisciplinaires. On a noté une prédominance féminine (56,04%). la plupart des patients étaient jeunes avec un pic de fréquence entre 20 ans et 39 ans soit 53,54%, ces données se rapprochent de celles de Njifou (52 ,09%) [1], d'Amara (49,29%) [2] et Noupoue (50,09%) [3]. La gratuité de la consultation et de l'ensemble des prestations médicales, jointe au caractère étendu à toute la population de Conakry, à l'effectif technique relativement élevé de l'HMCC et à la souplesse horaire, font que la population étudiée était une population assez représentative de la ville de Conakry. Néanmoins les résultats épidémiologiques de cette expérience ne peuvent être extrapolés à la Guinée Conakry.

### Spécificités pathologiques

**Pathologies infectieuses:** il ressort de notre étude, que les pathologies infectieuses étaient les plus fréquentes (54,51%) avec une prédominance des infections rhino-sinusiennes (20,09%), pharyngo-laryngées (19,98%) et des otites (13,96%). Cette prédominance des affections infectieuses était soulevée par Njifou (50,92%) [1], Gentillini (46,4%) [4] et Amara (48,43%) [2]. Ces chiffres élevés pouvaient s'expliquer par la mauvaise hygiène de vie, l'automédication et les conditions climatiques tropicales. Les infections rhino-sinusiennes peuvent être responsables de complications graves mais encore observées en Afrique subsaharienne. Ainsi, selon une série de 23 cas de sinusites aigues compliquées d'infections orbitaires observées au Cameroun, la sinusite était responsable de 17% de décès des patients. De même, elle était responsable de 14% de cellulites cervico-faciales avec 13% de décès selon une autre série [5]. Nous avons colligé 3 cas d'infections orbitaires à point de départ sinusien. Les affections infectieuses pharyngo-laryngées étaient dominées par les angines, les rhinopharyngites avec bien souvent des amygdales érythémateuses ou értyhémato-pultacées, sans qu′un avis médical ne soit demandé. En milieu tropical, le traitement antibiotique est systématique devant toute angine érythémateuse ou érythémato-pultacée jusqu’à l'âge de 25 ans, afin d’éviter la survenue du rhumatisme articulaire aigu. Les otites représentaient 13,96% de la pathologie infectieuse. Il s'agissait d'otites externes (65 cas), d'otites moyennes aigues (67 cas) et d'otites moyennes chronique (130 cas). L'otite externe présente des particularités en Afrique subsaharienne, l'otomycose y est plus fréquente que l'otite externe bactérienne [5].

**Figure 2:**
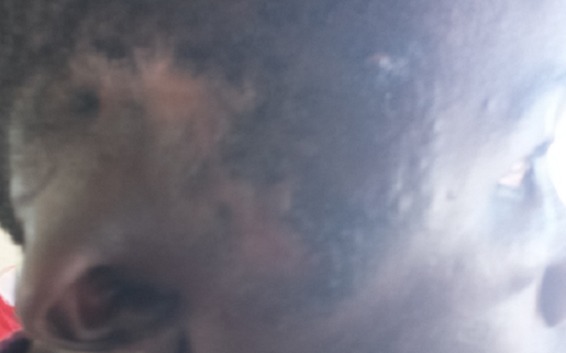
Mastoïdite aigue compliquant une otite moyenne chronique

La prévalence de l'otomycose dans notre série n’était pas soulevée par défaut d'examen mycologique. Selon les séries africaines, l'otite moyenne aigue est caractérisée par son taux élevé de complications [5]. Nous avons colligé 13 cas de perforations tympaniques otorrheiques et 2 cas de mastoïdites aigues compliquant des otites moyenne aigues négligées, essentiellement chez des enfants. L'otite moyenne chronique était la plus fréquente dans notre travail. Ogisi qualifiait l'otite moyenne chronique comme étant la maladie de l'oreille la plus fréquente dans les pays tropicaux [5]. Dans notre étude, il s'agissait le plus souvent d'otites moyennes chroniques suppuratives vues à un stade tardif. Le retard diagnostique en Afrique subsaharienne explique le nombre élevé de patients présentant une altération de l'audition ainsi que le taux élevé de complications [6]. On a observé 3 cas de mastoïdites aigues extériorisées (Figure 2) et 2 cas de paralysies faciales dont la cause était une otite moyenne chronique. Le recrutement ganglionnaire d'origine infectieuse était faible (0,48%).7 cas de tuberculose ganglionnaire étaient identifiés, parmi ces patients, 2 cas avaient une sérologie VIH (virus d'immunodéficience humaine) positive. La tuberculose associée à l'infection par le VIH a une place très particulière en zone tropicale. Les atteintes ganglionnaires cervicales sont plus précoces dans le déficit immunitaire et de présentation moins aiguë que les atteintes profondes, médiastinales ou abdominales [5].

**Les affections otologiques non infectieuses:** les affections otologiques non infectieuses entrainant surdité ou acouphènes étaient notées dans 18,96% des cas. Les surdités de perception y représentaient (86%), les surdités de transmission à tympan normal (9%) et les surdités mixtes (5%). Le diagnostic précis n’était pas posé, on pourrait retrouver dans ce groupe, des cas d'otospongiose, de presbyacousie, de surdité post-méningitique. Il existait en effet des difficultés non seulement dans l'exploration audiométrique (le CHU de Conakry ne possédait qu'un audiomètre tonale, absence d'impédancemètre, d'ottoemissions, de potentiels évoqués auditifs), mais aussi dans la prise en charge des patients sourds. Durant la période d’étude, ces difficultés se traduisaient par l'absence d'audioprothésistes et de structures d'accueil pour personnes sourdes dans toute la Guinée. La pathologie inflammatoire rhinosinusienne et pharyngée La pathologie inflammatoire rhinosinusienne et pharyngée représentait 18,01% des cas. La rhinite chronique était retrouvée dans 14,97%, ce chiffre est proche des 13% décrit par Amara [2]. Ce chiffre rapporté dans notre étude, pourrait s'expliquer par les conditions climatiques et surtout par le niveau de pollution élévé de la ville de Conakry (plus grande ville industrielle du pays, plus grand nombre de véhicules circulants, densité de la popullation élevée).

**Figure 3:**
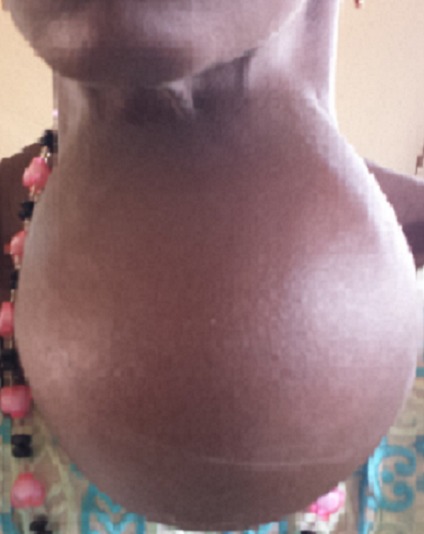
Goitre endémique chez une patiente de 46 ans

**Figure 4:**
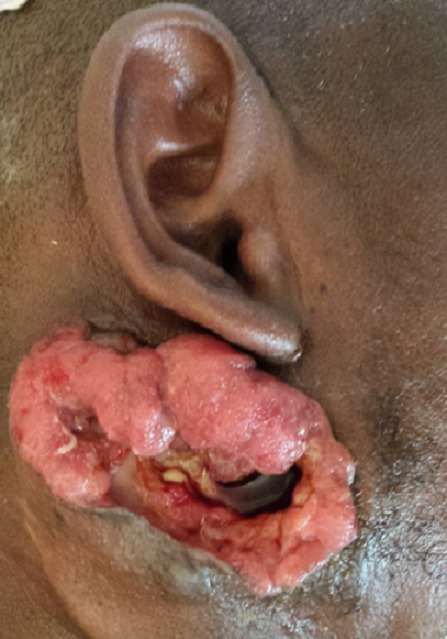
Récidive d'une tumeur parotidienne opérée

**La pathologie tumorale:** la pathologie tumorale représentait 6,34% des cas dans notre série. Dans la majorité des cas, il s'agissait de goitres multinodulaires endémiques (83 cas) (Figure 3). L'endémie goitreuse constitue un problème de santé très important pour le cas de la république de Guinée, avec un taux de prévalence nationale de 63,4%, ce taux élevé est expliqué par la carence en iode (faible teneur du sol en iode), les habitudes alimentaires (consommation de manioc, légumes goitrigenes) et la carence protidique [7]. Les tumeurs pharyngo-laryngés étaient retrouvés chez 7 cas (5 hommes et 2 femmes), l âge moyen était de 35 ans, souvent à des stades tardifs. En Afrique subsaharienne, les problèmes encourus sont surtout thérapeutiques, les auteurs rattachaient ces difficultés au bas niveau socio-économique de la population [5]. Dans notre série, la majorité de ces patients n'avaient pas les moyens de payer les frais d'une endoscopie sous anesthésie générale, voir une éventuelle intervention chirurgicale ou une radiothérapie. Les tumeurs des glandes salivaires étaient retrouvées chez 6 cas (4 hommes et 2 femmes), essentiellement de siège parotidien (4 cas) (Figure 4) et sous maxillaire (2 cas). La pathologie des glandes salivaires est une composante mineur de la carcinologie cervico-faciale en Afrique subsaharienne, ces tumeurs glandulaires représentaient 2,5% des cancers ORL [5]. La encore, comme pour les tumeurs pharyngo-laryngées, le diagnostic clinique ne posait pas de problèmes, cependant l'arsenal d'examens para-cliniques était limité, aucune cytoponction histologique n’était pas possible, encore moins une imagerie par résonnance magnétique pour les tumeurs parotidiennes.

**Les corps étrangers de la sphère ORL:** les corps étrangers de la sphère ORL étaient retrouvés chez 13 patients, le plus souvent des enfants de sexe masculin (11 cas). De nature diverse (cailloux, fragments d’éponge, fragments de craie, arrêté de poisson, boules de papier), ces corps étrangers siégeaient le plus souvent au niveau des fosses nasales (7 cas), l'oreille (4 cas) et le pharynx (2 cas). La particularité en guinée était le long délai d'admission, la majorité des patients étaient vus au delà de 24 heures. La fréquence des corps étrangers de la sphère ORL dans notre étude, reste relativement faible par rapport aux séries africaines [8].

**Figure 5:**
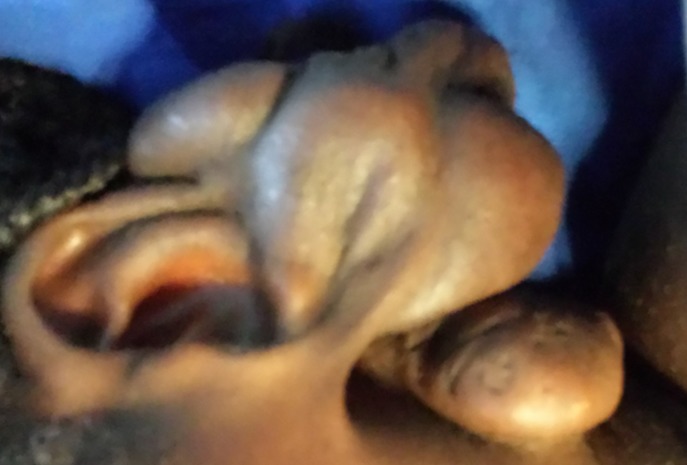
Chéloïde chez une patiente de 24 ans suite au piercing de l'oreille

**Les chéloïdes:** les chéloïdes étaient retrouvées chez 8 cas (0,43%). Chez l'homme, les traumatismes répétés lors des jeux ou des travaux et les rituels de scarifications étaient les causes habituelles, chez la femme, les chéloïdes étaient soit spontanées, soit dues le plus souvent au piercing des oreilles (Figure 5) et plus rarement à des scarifications. Cette fréquence rapportée dans notre série est relativement basse, comparée à d'autres séries [9].

**Figure 6:**
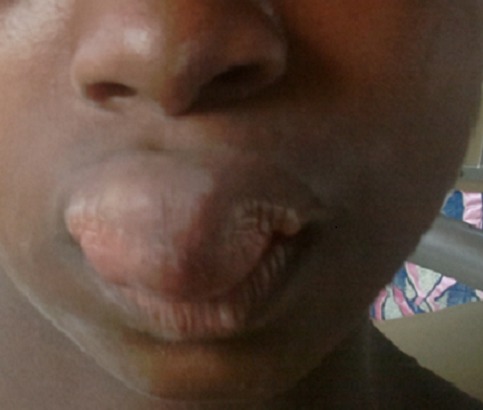
Malformation vasculaire labiale chez une fille de 12 ans

**La pathologie malformative:** la fréquence de la pathologie malformative dans notre série était de 0,37% de la pathologie ORL générale. Ce chiffre est proche du 0,43% rapporté par Njifou [1]. Nous avons trouvé 4 cas de fentes labiales, 2 cas de malformations vasculaires labiales (Figure 6) et 1cas d'aplasie de l'oreille.

**Figure 7:**
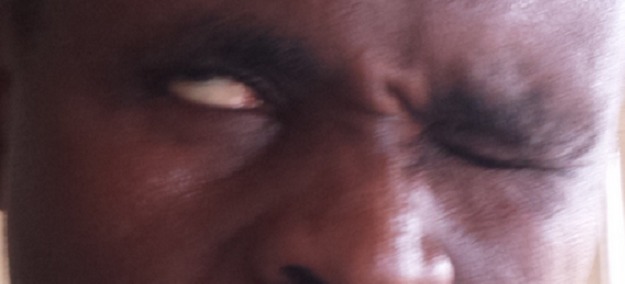
Paralysie faciale périphérique gauche Chez un patient de 45 ans

**Les paralysies faciales périphériques:** les paralysies faciales péripheriques en afrique subsaharienne connaissent un regain d'interet dans les publications médicales depuis l’épidémie du virus d'immunodéficience humaine(VIH). Le système nerveux est un organe cible, fréquemment atteint au cours de l'infection VIH, en particuliers le nerf facial [5]. Dans notre étude, nous avons colligé 4 cas de paralysies faciales (Figure 7), parmi eux un patient était infecté par le VIH. De ce fait la paralysie faciale constituerait un indicateur précoce de l'infection par le VIH dans le contexte africain.

**Regroupement topographique:** au plan topographique, nos résultats (Tableau 3) sont proches de ceux publiés par Njifou [1], par contre une étude faite au Nigéria par Eziyi [10], qualifiait les atteintes otologiques comme étant les plus Fréquentes (51,8%), suivis des atteintes rhino-sinusiennes (26%), puis des atteintes pharyngo-laryngés (15%) et des tumeurs de la tête et cou (7,2%).

## Conclusion

Cette mission humanitaire, nous a permis d'appréhender l’éventail de la pathologie ORL observée en Guinée Conakry. Cette dernière pose un problème de prise en charge diagnostique et thérapeutique, ce qui concourt à la sous-médicalisation de cette région d 'Afrique subsaharienne.
